# Anomalous Right Coronary Artery With Interarterial Course: Risk Stratification and Surgical Decision-Making Using Coronary Computed Tomography Angiography-Derived Fractional Flow Reserve

**DOI:** 10.7759/cureus.58885

**Published:** 2024-04-24

**Authors:** Ujjawal Kumar, Usman Aslam, David L Mancuso, Zain Khalpey

**Affiliations:** 1 Department of Cardiothoracic Surgery, HonorHealth, Scottsdale, USA; 2 School of Clinical Medicine, University of Cambridge, Cambridge, GBR; 3 Department of General Surgery, HonorHealth, Phoenix, USA

**Keywords:** fractional flow reserve (ffr), coronary computed tomography angiogram (cta), interarterial course, anomalous aortic origin of the right coronary artery, anomalous coronary artery origin, right coronary artery (rca)

## Abstract

An anomalous right coronary artery (RCA) takeoff, a rare congenital condition often characterized by an interarterial RCA course between the pulmonary artery and the ascending aorta, can lead to symptoms of angina pectoris (chest pain) or even sudden cardiac death (SCD) due to compression of the RCA, although most patients remain asymptomatic. In this case report, we highlight the utility of computed tomography angiography (CTA)-derived fractional flow reserve (FFR), a minimally invasive technique used to assess the hemodynamic significance of coronary lesions, in the risk stratification and surgical decision-making process for a 46-year-old female patient presenting with exertional dyspnea and an anomalous RCA takeoff with an interarterial course. The information obtained from this imaging modality was instrumental in determining that surgical repair did not need to be performed urgently and could be scheduled as an elective case in the future.

## Introduction

The coronary arteries' crucial role in providing myocardial perfusion means that any anomalies in their anatomy can result in potentially fatal consequences. The coronary arteries are named for each artery's specific distal perfusion territory, rather than their origin. Typically, the right coronary artery (RCA) arises from an ostium just below the sinotubular junction of the right sinus of Valsalva. From there, it courses in the right atrioventricular (AV) groove, providing branches to the right ventricular free wall and extending to the acute margin of the heart. The distal territory of the RCA varies and can extend posteriorly as far as the obtuse margin of the heart. In approximately 90% of patients, the RCA supplies the posterior descending coronary artery branch at the crux of the heart, perfusing the AV node and the posterior aspect of the interventricular septum [1].

Coronary artery anomalies are a group of congenital disorders characterized by abnormal origin, course, or termination of one or more coronary arteries. The prevalence of coronary artery anomalies ranges from 0.2% to 1.2% in the general population [2]. Among these anomalies, an anomalous RCA takeoff from the left sinus of Valsalva with an interarterial course between the aorta and pulmonary artery is a rare condition, occurring in approximately 0.1-0.3% of patients undergoing coronary angiography [3,4]. Although most patients with this anomaly remain asymptomatic, a small percentage may experience symptoms such as angina pectoris (chest pain) or even sudden cardiac death (SCD) due to compression of the RCA as it runs between the two large central arteries [5,6]. The significance of this is shown by the fact that up to 35% of sudden deaths in those under 35 are thought to be due to non-atherosclerotic coronary artery disease [7]. The mechanism of ischemia in patients with an interarterial course of the RCA is thought to be related to the compression of the anomalous vessel during exercise when there is increased cardiac output with diastolic dilatation of the aorta and pulmonary artery [8]. While there are established trends between young athletes with this anomaly and the incidence of SCD [9], there is still a proverbial gray area regarding interventional decision-making in older, non-athletic individuals.

Computed tomography angiography (CTA) has emerged as a valuable non-invasive imaging modality for the diagnosis and evaluation of coronary artery anomalies [10]. Furthermore, CTA-derived fractional flow reserve (FFR) is a reliable tool for assessing the hemodynamic significance of coronary lesions, providing valuable information for clinical decision-making [11]. It can also be applied to other causes of flow limitation such as external compression in cases of an interarterial coronary artery course [12]. In this case report, we present a 46-year-old female patient with an anomalous RCA takeoff from the left sinus of Valsalva and an interarterial course, highlighting the utility of CTA-derived FFR in risk stratification and surgical decision-making.

## Case presentation

**Figure 1:**
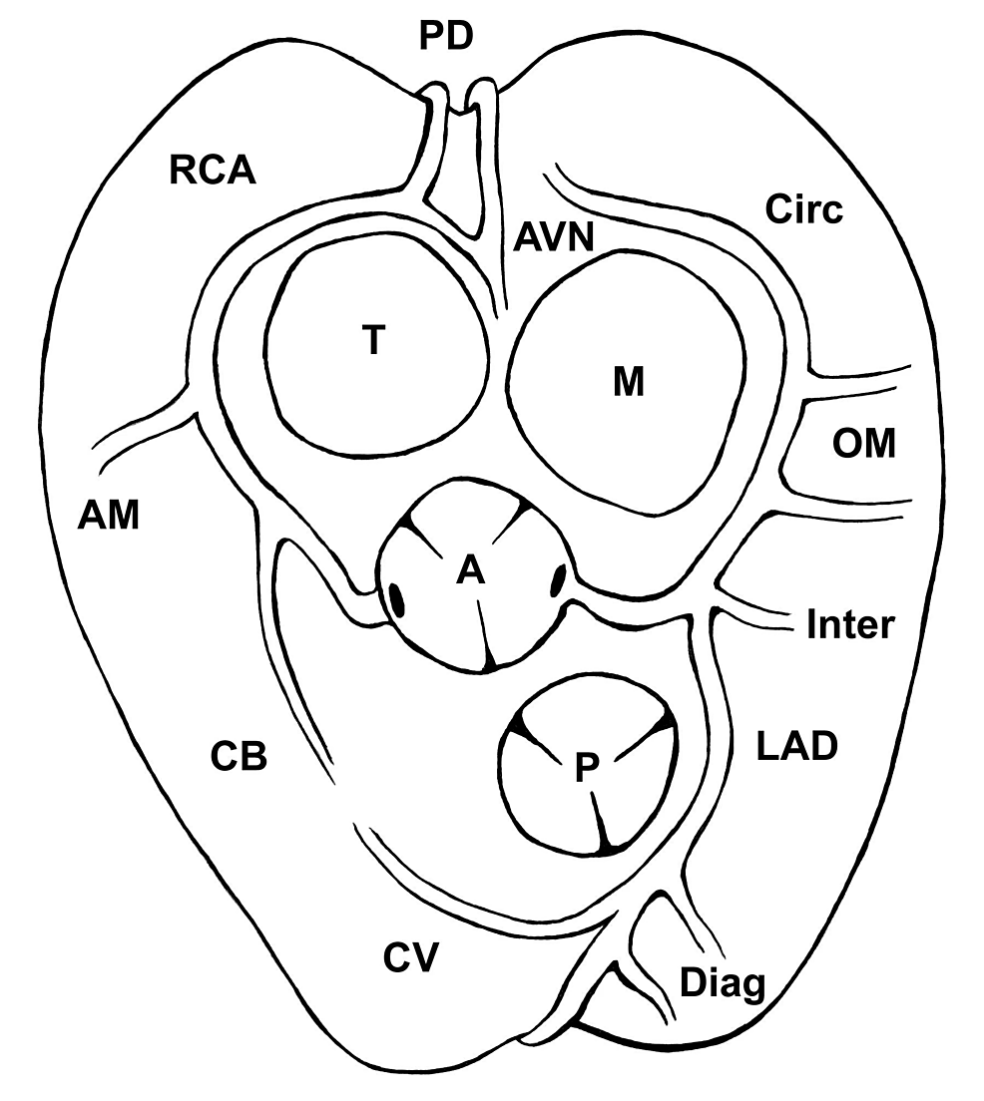
The normal anatomy of the coronary arteries, viewed from above with the atria removed. Given the rarity of any anomalous coronary artery anatomy and no family history of anomalous coronary anatomy, it was expected that this patient would have normal coronary anatomy as shown above. A: aortic valve; P: pulmonary valve; T: tricuspid valve; M: mitral valve; RCA: right coronary artery; AM: acute marginal branch of the right coronary artery; CB: conus branch of the right coronary artery; PD: posterior descending branch; AVN: atrioventricular nodal branch; Circ: circumflex coronary artery; OM: obtuse marginal branches of circumflex coronary artery; LAD: left anterior descending coronary artery; Diag: diagonal branches of the left anterior descending coronary artery; Inter: intermedius branch of the left coronary artery

A 46-year-old female presented to her cardiologist's office with a chief complaint of dyspnea on exertion. Her medical history was significant for hyperlipidemia and pharmacologically controlled hypertension. Family history revealed that her father had experienced SCD at age 61 and her mother passed away from acute thoracic aortic dissection (Stanford type A) at age 64. Due to insurance denial of provocative stress testing, she underwent coronary CTA for assessment and risk stratification. This was expected to show normal coronary anatomy (Figure 1), with some degree of atherosclerotic changes resulting in myocardial ischemia and the chest pain that she was experiencing.

**Figure 2:**
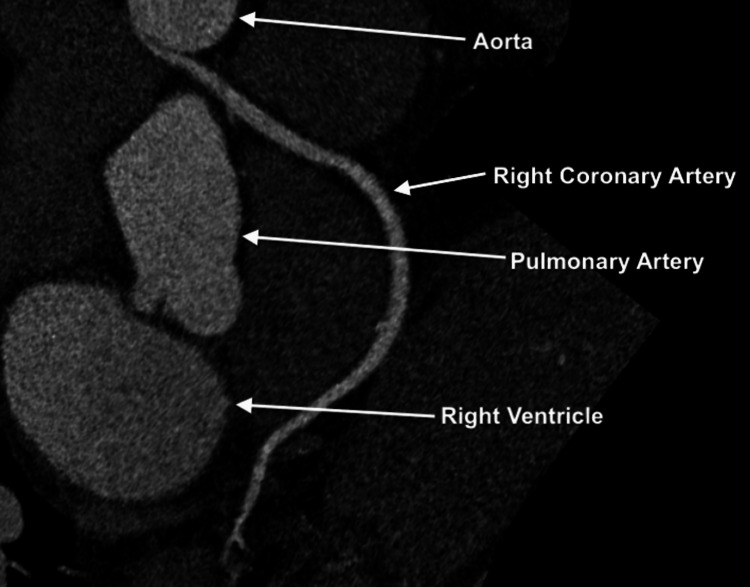
CTA image showing the anomalous origin of the RCA in this patient, as well as the interarterial course. CTA image showing the interarterial course of the RCA. The anomalous RCA is seen, arising from the left sinus of Valsalva and coursing between the aorta and the PA. Note the oblique origin and the intramural course within the aortic wall, all factors compromising coronary blood flow. CTA: computed tomography angiography; RCA: right coronary artery; PA: pulmonary artery

**Figure 3:**
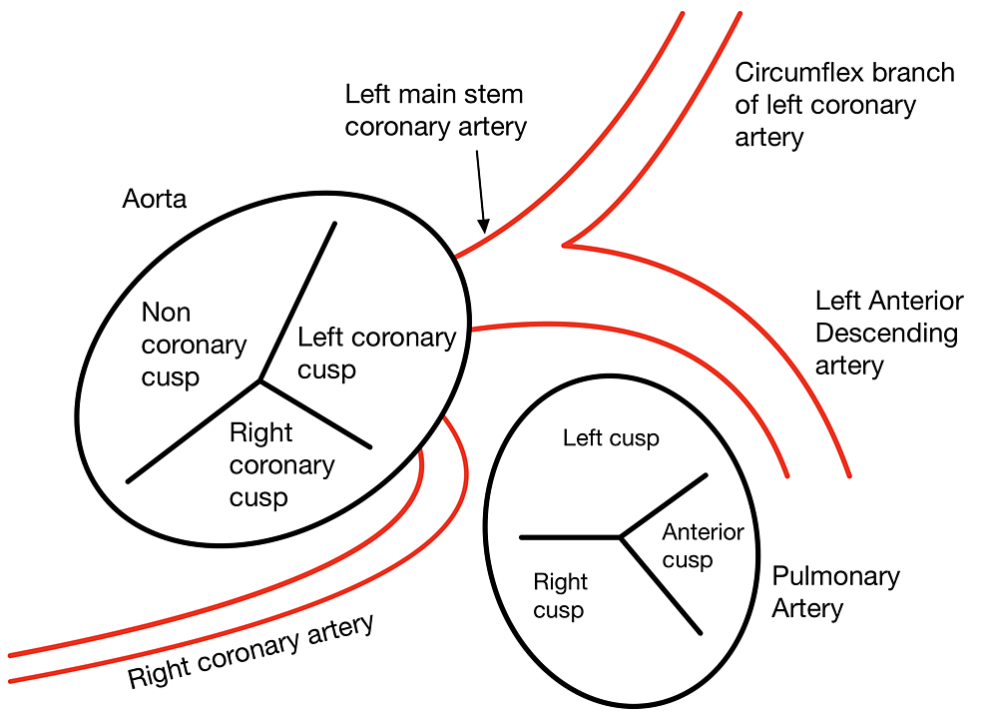
Representation of an anomalous origin of the RCA from the left sinus of Valsalva and an interarterial course, as seen in this patient. RCA: right coronary artery

The coronary CTA imaging showed no hemodynamically significant atherosclerotic lesions; however, an anomalous RCA takeoff from the left coronary cusp with an interarterial course between the pulmonary artery and the ascending aorta was noted (Figure 2). This "malignant" course (depicted in Figure 3) predisposes the patient to distal myocardial ischemia due to extrinsic coronary artery compression by the great vessels, which expand during exercise and show diastolic dilatation. Subsequent PET myocardial perfusion stress testing revealed no evidence of reversible ischemia, infarction, or any perfusion defects. The patient was referred for outpatient cardiothoracic surgical evaluation to assess the need for definitive surgical treatment.

**Figure 4:**
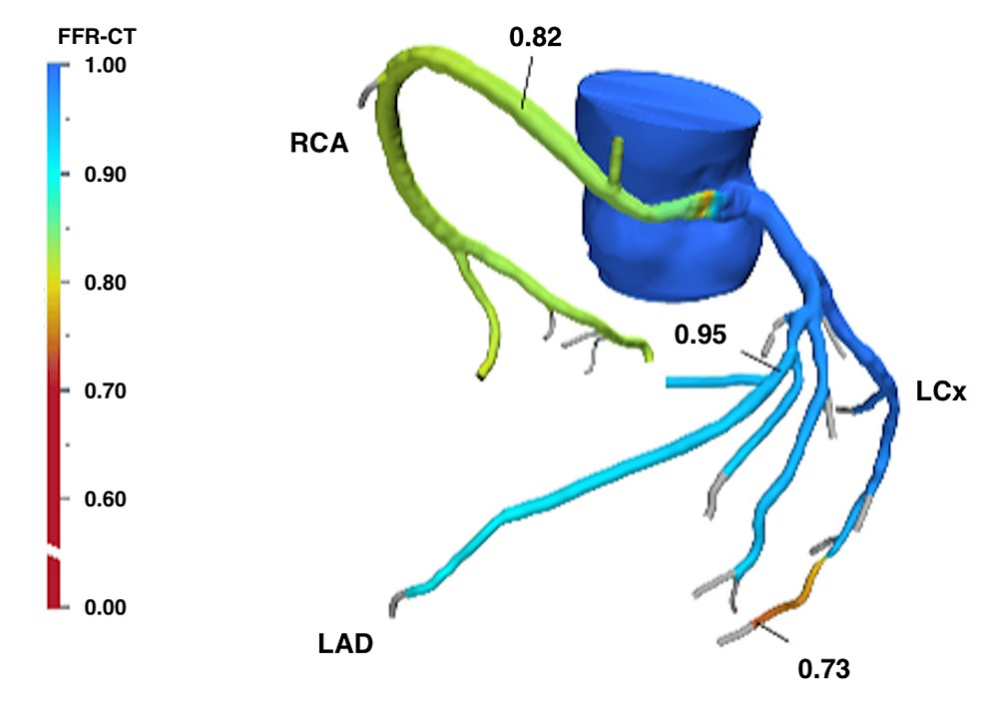
CTA-derived FFR investigation for this patient does not show a significant coronary lesion in the RCA warranting urgent surgery. Results from the CTA-derived FFR investigation showed the hemodynamically insignificant coronary lesion with an FFR value of 0.82 in the RCA, measured 2 cm distal to the region of proximal RCA narrowing. CTA: computed tomography angiography; FFR: fractional flow reserve; RCA: right coronary artery; LAD: left anterior descending; LCx: left circumflex

Upon presentation for cardiothoracic surgical evaluation, the patient's physical exam was unremarkable, but her complaints of exertional dyspnea persisted. Although the negative stress test findings would typically contraindicate surgery, the presence of persistent anginal-like symptoms warranted further investigation. A coronary CTA with FFR was ordered to quantify RCA flow and determine the urgency of surgical intervention necessary due to any potential hemodynamically significant RCA compression. The CTA imaging with FFR revealed an RCA diameter of 1.8 mm as it coursed between the left and right ventricular outflow tracts, subsequently measuring 4.5 mm immediately to the right of the aorta. The RCA demonstrated an FFR CT value of 0.82, measured 2 cm distal to the region of proximal RCA stenosis (Figure 4).

Based on these findings and established clinical conventions, the cardiothoracic surgical multidisciplinary team determined that the patient could safely delay surgery. The patient was advised to return for follow-up in six months with updated cardiac stress testing to reassess the need for surgical intervention. Regular follow-up intervals will be maintained to ensure proper surveillance and monitoring, which is crucial in preventing potential future morbidity or mortality associated with this anomalous coronary anatomy. The patient was also advised to avoid significantly strenuous activity in the interim to minimize the risk of sudden cardiac events, as is recommended for all young individuals with untreated anomalous coronary artery anatomy, particularly those with a malignant course.

## Discussion

Anomalous coronary artery anatomy is a rare but potentially life-threatening condition that can lead to myocardial ischemia, arrhythmias, and SCD. In this case, the patient presented with exertional dyspnea and was found to have an anomalous RCA with a malignant interarterial course between the aorta and pulmonary artery. This particular anomaly is considered high-risk due to the potential for compression of the RCA during exercise, leading to ischemia and adverse cardiac events [4]. Using CTA with FFR played a pivotal role in assessing the hemodynamic significance of the RCA compression and guiding surgical decision-making. CTA allows for detailed visualization of the coronary artery anatomy, including the origin, course, and termination of the vessels [10]. The addition of FFR provides valuable functional information about the hemodynamic significance of any stenosis or compression [13].

In this case, the FFR value of 0.82 indicated that the RCA compression was not currently hemodynamically significant, allowing the surgical team to safely delay intervention and opt for close monitoring and follow-up. According to the Radiological Society of North America guidelines, an FFR value ≥0.80 excludes lesion-specific ischemia with a negative predictive value of >95% [14]. A study by Pijls and colleagues investigated the need for revascularization among 45 individuals with varying coronary FFR values. Of the 24 patients with an FFR >0.75, 21 tested negative for reversible myocardial ischemia. No revascularization procedures were performed for these 24 individuals, and none required revascularization over the subsequent 14 months of follow-up [15].

One of the major advantages of CTA-FFR over traditional catheter angiography with FFR is its non-invasive nature. CTA-FFR can be performed using data obtained from a standard coronary CTA, without the need for additional invasive procedures or the administration of adenosine to induce hyperemia [11]. This reduces the risk of complications associated with invasive procedures and allows for a more comprehensive assessment of the entire coronary artery tree. Additionally, CTA-FFR has been shown to have good diagnostic accuracy compared to invasive FFR, with several studies demonstrating high sensitivity and specificity [13,16].

The management of patients with anomalous coronary arteries remains challenging, as there are no clear guidelines for when to intervene surgically. Factors such as the patient's age, symptoms, and the specific anatomic features of the anomaly must be considered. In general, surgical repair is recommended for patients with documented ischemia, arrhythmias, or aborted SCD [17]. However, the decision to intervene in asymptomatic patients or those with equivocal findings is less clear. Some studies suggest that surgical repair should be considered in all patients with a malignant interarterial course, regardless of symptoms, due to the unpredictable nature of the condition [18]. Others argue for a more conservative approach, reserving surgery for those with clear evidence of ischemia or hemodynamic compromise [19].

In this case, the patient's persistent symptoms despite negative stress test results warranted further investigation with CTA-FFR. The findings allowed for a personalized approach to management, balancing the risks and benefits of surgical intervention. By opting for close surveillance and follow-up, the surgical team acknowledged the potential for future complications while avoiding unnecessary surgery at present. While the advice to not engage in strenuous activity may limit the patient's range of activities, this was a shared decision that was made with patient input, as it was judged that the risk of adverse an event and the ability to delay surgery for now justified this limitation. These suggestions were made under the assumption that the patient's symptoms were attributable to partial compression of the RCA during strenuous activity and supported by the absence of other flow-limiting coronary lesions.

This case highlights the importance of a comprehensive diagnostic approach in patients with anomalous coronary artery anatomy. CTA with FFR provides valuable anatomical and functional information, enabling a more informed decision-making process. In patients with persistent symptoms despite negative stress test results, further investigation with advanced imaging modalities like CTA-FFR can help identify those who may benefit from surgical intervention and those who can be safely managed with close surveillance. To promote these findings, additional studies should be conducted to look at varying CTA-derived FFR values for anomalous RCAs as well as long-term outcomes based on the decisions made with pre-existing FFR criteria in mind and comparison, with already established invasive modalities.

## Conclusions

Anomalous RCA with a malignant interarterial course is a rare congenital anomaly that can lead to potentially fatal consequences, including myocardial ischemia and SCD. The management of these patients remains challenging, as there are no clear guidelines for when to intervene surgically. CTA with FFR is a valuable diagnostic tool for assessing the hemodynamic significance of coronary lesions and guiding surgical decision-making in these patients. This case report demonstrates the clinical utility of CTA-FFR in the risk stratification and management of a patient with anomalous RCA. By providing detailed anatomical and functional information, CTA-FFR allowed for a personalized approach to management, balancing the risks and benefits of surgical intervention. The findings supported a decision to delay surgery and opt for close surveillance while acknowledging the potential for future complications.

As our understanding of anomalous coronary artery anatomy continues to evolve, we must refine our diagnostic and management strategies to optimize patient outcomes. This case underscores the importance of a comprehensive diagnostic approach, utilizing advanced imaging modalities like CTA-FFR to guide decision-making. It also highlights the need for ongoing research to better define the natural history of these anomalies and establish clear guidelines for surgical intervention. By continuing to advance our knowledge in this area, we can improve the care of patients with anomalous coronary artery anatomy and reduce the risk of SCD in this population.

## References

[1] Laird RJ, Irwin S (2004). Chapter 1 - cardiovascular structure and function. Cardiopulmonary Physical Therapy (Fourth Edition).

[2] Angelini P, Velasco JA, Flamm S (2002). Coronary anomalies: incidence, pathophysiology, and clinical relevance. Circulation.

[3] Yamanaka O, Hobbs RE (1990). Coronary artery anomalies in 126,595 patients undergoing coronary arteriography. Cathet Cardiovasc Diagn.

[4] Cheezum MK, Liberthson RR, Shah NR, Villines TC, O'Gara PT, Landzberg MJ, Blankstein R (2017). Anomalous aortic origin of a coronary artery from the inappropriate sinus of Valsalva. J Am Coll Cardiol.

[5] Taylor AJ, Rogan KM, Virmani R (1992). Sudden cardiac death associated with isolated congenital coronary artery anomalies. J Am Coll Cardiol.

[6] Greet B, Quinones A, Srichai M, Bangalore S, Roswell RO (2012). Anomalous right coronary artery and sudden cardiac death. Circ Arrhythm Electrophysiol.

[7] Basso C, Corrado D, Thiene G (2001). Congenital coronary artery anomalies as an important cause of sudden death in the young. Cardiol Rev.

[8] Angelini P (2007). Coronary artery anomalies: an entity in search of an identity. Circulation.

[9] Maron BJ, Doerer JJ, Haas TS, Tierney DM, Mueller FO (2009). Sudden deaths in young competitive athletes: analysis of 1866 deaths in the United States, 1980-2006. Circulation.

[10] Gräni C, Buechel RR, Kaufmann PA, Kwong RY (2017). Multimodality imaging in individuals with anomalous coronary arteries. JACC Cardiovasc Imaging.

[11] Nørgaard BL, Leipsic J, Gaur S (2014). Diagnostic performance of noninvasive fractional flow reserve derived from coronary computed tomography angiography in suspected coronary artery disease: the NXT trial (Analysis of Coronary Blood Flow Using CT Angiography: Next Steps). J Am Coll Cardiol.

[12] Tesche C, De Cecco CN, Albrecht MH (2017). Coronary CT angiography-derived fractional flow reserve. Radiology.

[13] Driessen RS, Stuijfzand WJ, Raijmakers PG (2018). Effect of plaque burden and morphology on myocardial blood flow and fractional flow reserve. J Am Coll Cardiol.

[14] Rajiah P, Cummings KW, Williamson E, Young PM (2022). CT fractional flow reserve: a practical guide to application, interpretation, and problem solving. Radiographics.

[15] Pijls NH, De Bruyne B, Peels K, Van Der Voort PH, Bonnier HJ, Bartunek J Koolen JJ, Koolen JJ (1996). Measurement of fractional flow reserve to assess the functional severity of coronary-artery stenoses. N Engl J Med.

[16] Renker M, Schoepf UJ, Wang R (2014). Comparison of diagnostic value of a novel noninvasive coronary computed tomography angiography method versus standard coronary angiography for assessing fractional flow reserve. Am J Cardiol.

[17] Mery CM, De León LE, Molossi S (2018). Outcomes of surgical intervention for anomalous aortic origin of a coronary artery: a large contemporary prospective cohort study. J Thorac Cardiovasc Surg.

[18] Mainwaring RD, Reddy VM, Reinhartz O (2011). Anomalous aortic origin of a coronary artery: medium-term results after surgical repair in 50 patients. Ann Thorac Surg.

[19] Agrawal H, Mery CM, Krishnamurthy R, Molossi S (2017). Anatomic types of anomalous aortic origin of a coronary artery: a pictorial summary. Congenit Heart Dis.

